# Short-Term Music Training Enhances Spectral Resolution for Prelingually Deafened Children with Cochlear Implants

**DOI:** 10.3390/audiolres16030073

**Published:** 2026-05-13

**Authors:** Chi Yhun Lo, Valerie Looi

**Affiliations:** 1 Department of Linguistics, Macquarie University, Sydney, NSW 2109, Australia; 2 Australian Institute of Health Innovation, Macquarie University, Sydney, NSW 2109, Australia; 3 Performance and Expertise Research Centre, Macquarie University, Sydney, NSW 2109, Australia; 4 Parents of Deaf Children, Macquarie University, Sydney, NSW 2109, Australia; 5 Spiral Therapeutics, San Francisco, CA 94080, USA

**Keywords:** spectral resolution, cochlear implants, children, music, music training, SMRT

## Abstract

Background/Objectives: Spectral resolution is strongly associated with speech perception for adult cochlear implant users, but the developmental trajectory of spectral resolution in childhood is more complex and far less understood. Music-based training presents a unique opportunity to address this gap, as musical stimuli feature spectral complexity and fine frequency cues which map to spectral resolution. This study explored if a 12-week music-based intervention could support better spectral resolution in children with cochlear implants. Methods: Twelve children with cochlear implants participated in this longitudinal, repeated-measures study. The music training intervention consisted of group-based in-person music therapy and a take-home music app. Participants (six boys, six girls; *M* age = 7.3 years) were pseudo-randomized into an immediate training group (*n* = 4) or delayed-start waitlisted group (*n* = 8). Inclusion criteria required bilateral moderate-to-profound sensorineural hearing loss, prelingual device fitting, and consistent bilateral device use. Eight children had bilateral CIs and four were bimodal listeners. Results: Spectral resolution perception was significantly enhanced after participating in the music intervention with a mean increase of 2 rpo, *F*(3, 10.7) = 3.859, *p* = 0.017. Previous engagement with music and age were not associated with spectral resolution. Conclusions: Despite the known limitations of CIs on spectral resolution, the results of this study indicate that music training can improve spectral resolution perception in children using CIs.

## 1. Introduction

Cochlear implants (CIs) can provide early access to sound for children with severe-to-profound hearing loss (HL), supporting the development of speech, language, and communication [1]. Despite these benefits, outcomes are heterogenous, and real-world, complex listening environments such as noisy or multi-talker situations are a common challenge [2]. These challenges reflect complex interactions between peripheral and central auditory processes, cognitive maturation, and the child’s broader access to language and communication strategies [3]. One percept implicated in these processes is spectral resolution—the ability to detect, differentiate, and integrate frequency-based cues within the acoustic signal [4,5].

Spectral resolution allows listeners to distinguish fine frequency differences in formant structure, harmonic spacing, and spectral envelopes—cues that are foundational to vowel identity, consonant place of articulation, and phonemic contrast [4]. The development of spectral resolution is related to both frequency resolution and sensitivity to intensity modulation. The former refers to the tuning of one’s auditory filters and the ability to resolve the spectral place of the signal [6], whereas the latter is the ability to compare intensity cues across frequencies and perceive difference in the intensity of the spectral component of the signal [7,8]. However, research has shown that CI users have diminished frequency resolution and intensity modulation skills, in part related to the technology itself (e.g., bandpass filtering, limited electrode coverage of the cochlea, current spread in the cochlea, frequency-to-place mismatch to name only a few; see [9,10] for more detail), as well as psychoacoustic limitations associated with sensorineural HL [11]. These constraints result in a blurred or “smeared” frequency representation, reducing access to the spectral information that supports robust speech understanding [12].

In adult CI users, the relationship between spectral resolution and speech perception is well established. Individuals with better spectral resolution consistently demonstrate better performance on word recognition, vowel identification, and speech-in-noise (SIN) tasks [13,14]. However, for children with CIs, the association between spectral resolution and speech perception is mixed [15]. Whilst children with CIs show poorer spectral resolution compared with their typical-hearing (TH) peers [5], its association with speech outcomes is inconsistent. Some pediatric cohorts demonstrate moderate associations between spectral modulation sensitivity and word or vowel recognition [16,17]. On the other hand, a recent cross-sectional study found that spectral resolution was not associated with speech perception in quiet or in noise [18].

Jahn et al. conducted a review of spectral resolution development in children with TH and CIs [15]. In TH children, performance was correlated with age and maturation occurred between the ages of 8–11 years. However, for children with CIs, spectral resolution was not correlated with chronological age, nor age of implantation (i.e., hearing age) [5], and maturation continued well into teenage years (12–14 years), indicating a delay relative to TH peers [3,19,20]. It may be that there is an upper limit to how much spectral resolution a child can perceive through a CI, with maturation and development exerting minimal influence beyond this technological limit. Whether experience with the CI correlates to spectral ripple performance is still under investigation with conflicting results to date in the limited number of studies available [15]. These mixed findings necessitate several considerations. The auditory development of children with CIs, particularly those who were prelingually implanted, is significantly different to their TH peers. Aside from the obvious difference in the trajectory of their auditory development and the lack of a ‘normal hearing’ auditory template to compare sounds back to, children with CIs likely rely disproportionately on temporal-envelope cues rather than spectral resolution, an effective cue-weighting adaptation due to the reduced spectral resolution provided by CIs [21].

Another factor that could contribute to the variability in spectral resolution outcomes is experience with formal music training and informal music engagement. Formal music training incorporates music lessons, orchestra/band/choir practices, and music education sessions often led by an individual where the content is somewhat pre-planned and there is usually a learning objective. Informal music engagement would include activities such as singing at home or in the car with parents/family, moving/dancing to music playing in the background, going to a concert, or singing along to the radio or TV. Enjoyment and appreciation of music tend to be the goals as opposed to learning a specific musical skill, or a pre-determined learning objective.

Given that fine frequency discrimination is an inherent component of melodic perception, it would seem reasonable to consider that a participant who has had more music training or experience may have more fine-tuned spectral resolution than a participant with minimal music exposure. The review paper by Jahn et al. [15] discussed the underlying reduced processing efficiency and immaturity of the auditory processing system for a child with CIs when compared to a child with TH, and how the trajectory of development is highly variable, protracted, and affected by many internal and external factors. However, one unaddressed question is whether music training/participation may facilitate and/or expedite this maturational process. For example, in a retrospective analysis of 209 adult CI users, previous formal music training at high school or later was a significant predictor of performance on music-based tasks where no lyrics were involved (e.g., pitch or timbre perception) as the listener would be more reliant on spectral information when the linguistic cues were absent [22]. A recent study by Aksu et al. [23] utilized a music-integrated phonological awareness program for children with CIs. Encouragingly, both phonological awareness and spectral resolution were significantly improved post-intervention with the authors suggesting that the integration of music into the program specifically supported spectral resolution development.

Only a few adult and pediatric CI studies have considered spectral resolution as an outcome of interest, providing mixed and limited evidence that it can be trained and enhanced [24,25,26]. In these studies, real-world musical or linguistic stimuli were used as training materials and the generalization of this to spectral ripple perception was evaluated (i.e., participants were not trained with spectral ripples with the aim of improving spectral perception). However, two studies which utilized spectral ripples as the primary stimuli of the auditory training with the adult CI users found that this task-specific training significantly improved their spectral resolution [27,28].

Music-based training presents a unique opportunity to explore whether spectral resolution can be improved in CI recipients. Musical stimuli are typically rich in spectral complexity and require listeners to engage with fine frequency discrimination tasks which maps directly to spectral resolution. The OPERA hypothesis posits that music training promotes adaptive plasticity through overlapping neural resources, perceptual demands that require more precision than speech perception, emotional engagement, intensive repetition, and sustained attention [29,30]—features that are well suited to supporting auditory learning in children [31]. Notably, music-based interventions have demonstrated auditory benefits (primarily with SIN perception) in both adult and pediatric cohorts with TH, deaf, and hard-of-hearing populations [26,28,32,33,34,35,36,37], underscoring their potential benefit.

In summary, whilst existing research has shown that spectral resolution is strongly associated with speech perception for adult CI users, the developmental trajectory of spectral resolution development in childhood, and factors that impact on this, is more complex and far less understood. Critically, the absence of targeted auditory- or music-based interventions raises the question as to whether spectral resolution can be improved or its developmental trajectory enhanced. Addressing this gap is essential to both our theoretical understanding and informing subsequent (re)habilitation approaches that may meaningfully enhance communication, educational and quality-of-life outcomes for children with CIs. Thus, if music-based training can enhance spectral processing—or even facilitate more efficient use of the limited spectral information available through CIs—it may help children achieve greater perceptual, musical and communication outcomes, particularly in complex listening environments such as SIN.

In this study, a secondary data analysis was conducted on data from a previously reported study [26,38]. The original trial was a 12-week music-based intervention for children with HL (utilizing CIs and/or hearing aids), with the main findings being improved SIN perception and quality-of-life outcomes post training. This current manuscript reports on a subset from that cohort—children using either bilateral CIs or a bimodal configuration. This reduces one factor of variance by ensuring each participant received input from a CI and electrical stimulation of hearing in at least one ear. Given the limited evidence for developmental change in spectral resolution amongst pediatric CI users and the absence of studies examining training effects on this perceptual domain, a dedicated re-analysis of these data is both timely and warranted, particularly given the wealth of research reporting that spectral resolution is negatively impacted by the electrical stimulation of hearing from a CI [9,39]. Furthermore, spectral resolution testing is a non-linguistic assessment, meaning it can be used across different countries, languages, and population types irrespective of language ability or language spoken. This provides significant clinical and research value in that, unlike speech perception tests which are language- and even accent-specific, with different tests required for different countries/languages/populations, the SMRT can be used to assess and compare between different population types.

The aim of this study was to explore if music-based training could support the perception of spectral resolution for children with CIs. Based on the preceding literature, we propounded the following three hypotheses:

**H1.**  *Participation in the 12-week music intervention will be associated with better spectral resolution*.

**H2.**  *Prior formal and informal music engagement will be associated with better spectral resolution*.

**H3.**  *Chronological age and hearing age will be associated with better spectral resolution*.

As a secondary aim, we investigated if chronological age and hearing age were associated with formal or informal music engagement in an exploratory manner.

## 2. Materials and Methods

This study was part of a larger study, the details of which are published [26], and the overall methodology and participants were essentially as reported in that paper. A short summary of this is provided below, along with the specific details of the spectral ripple test stimuli and procedure that is novel to this paper.

### 2.1. Overall Study Design

This study adopted a longitudinal, repeated-measures, waitlist design lasting approximately 9 months. Twelve children with CIs were recruited and participated in a baseline testing session (Baseline 1). They were subsequently pseudo-randomized (for those who had specific timing/familial needs due to the study length) into either the music training group (Group 1, *n* = 4) or the waitlist group (Group 2, *n* = 8). Group 1 commenced with the 12-week music training program straight away, with Group 2 commencing training 12 weeks later. Due to the staggered-start design, a double-baseline approach was used; the second baseline was 1 week after Baseline 1 for Group 1, and 12 weeks after Baseline 1 for Group 2. For both groups, a mid-training assessment was conducted half-way through the training (week 6), a post-training assessment at training completion (week 12), and a follow-up assessment 12 weeks after training finished to measure retention (follow-up).

### 2.2. Participants

Six boys and six girls (age: *M* = 7.3 years; *SD* = 1.1; range = 6–9.2 years) were recruited for the study. Inclusion criteria were as follows:

- Aged between 6:0 and 9:11 years at enrolment.
- Bilateral moderate-to-profound sensorineural HL (consistent with the pediatric audiological criteria for a CI in Australia).
- Prelingual onset of hearing loss, with hearing aid(s) and/or CI(s) fitted < age 3.5 years.
- Bilateral hearing device use (i.e., bilateral CI or CI with contralateral hearing aid).
- Consistent full-time users of their hearing device(s).
- No additional impairments that would prohibit the completion of the task requirements (e.g., significant intellectual or visual impairment, diagnosed syndrome, etc.).

There were 8 bilateral CI recipients and 4 bimodal listeners. Of the 12 participants, nine commenced music training, with seven completing all testing sessions (two withdrawals occurred due to a medical reason and migration overseas which affected and reduced the statistical power of the follow-up assessment). See Table 1 for more details.

### 2.3. Materials

#### 2.3.1. Spectral-Temporally Modulated Ripple Test

The Spectral-Temporally Modulated Ripple Test (SMRT) Version 1.1 [40] was used to test spectro-temporal modulation detection. The SMRT is a non-linguistic, psychoacoustic measure that evaluates a person’s ability to discriminate spectral modulations. The rate of the spectral modulation (i.e., number of spectral peaks and troughs) within each stimulus octave is referred to as ‘ripples per octave’ (rpo). The SMRT uses 500 ms duration non-harmonic tone complexes with 202 equal-amplitude pure-tone frequency components spaced every 1/33.33 of an octave from 100 to 6400 Hz. Each tone has a 100 ms onset/offset linear ramp generated with a 44.1 kHz sampling rate.

A three-alternative forced choice (3-AFC) task response is used in which two choices were the reference stimuli at 20 rpo with the third being the target stimulus, initially presented at 0.5 rpo and progressing with 0.2 rpo step sizes in a 1-up/1-down adaptive procedure. Threshold is calculated from the results of the last 6 of 10 reversals.

#### 2.3.2. The Role of Music in Families Questionnaire

The Role of Music in Families Questionnaire (RMFQ) was developed to evaluate the role of music in families of children with HL and their general attitudes and level of engagement with music [41,42]. It comprised seven broad sections: General Demographic Information, Childhood Music Participation and Experiences, Attitudes and Reactions to Music, Resources for Child Regarding Music, Overall Importance of Music in Your Household and Family, Child’s Music Listening Preferences, and Future Perspective.

One section of the RMFQ (Childhood Music Participation and Experiences) was used in this study to appraise the level of formal music participation and experience each participant had received prior to commencement of this study. A ‘formal music participation’ score was calculated on the basis of duration (in terms of years) multiplied by its frequency (1 = less often than monthly, 2 = once a month, 3 = 2–3 times a month, 4 = once a week, 5 = 4–6 times a week, 6 = 2–3 times a week, and 7 = daily), divided by the total number of categories (*n* = 6) that assessed activities: music lessons, singing groups, instrumental groups, special children’s programs, dance classes, and group-based music classes. As an example, 1 year of weekly piano lessons equates to: 1 (year) × 4 (frequency, weekly) ÷ 6 (categories) = 0.7. The same approach was used to calculate an ‘informal music participation’ score, but as this featured nine categories, score calculation was divided by 9. As an example, 2 years of daily family musical activities equates to: 2 (years) × 7 (frequency, daily) ÷ 9 (categories) = 1.6.

Formal and informal categorization was as follows:

- Formal activities (*n* = 6): Music lessons, singing groups, instrumental groups, special children’s music programs, dance classes, and other organized music programs or activities.
- Informal activities (*n* = 9): Listening to music informally, social music activities, musical videos, family musical activities, online music training or games, independent music exploration, creating/making up songs or music performances during play, dancing informally, and music concerts.

### 2.4. Procedure

#### 2.4.1. The Role of Music in Families Questionnaire

The RMFQ was used as a measure of music engagement prior to enrolling in the music intervention. This questionnaire was completed by the parent/guardian whilst their child completed their first baseline test session.

#### 2.4.2. Spectral-Temporally Modulated Ripple Testing

All testing occurred in an acoustically treated sound booth with an experimental tester present to observe participant compliance. The SMRT provides three test response options which were displayed and inputted via a touchscreen monitor. SMRT stimuli were calibrated to 65 dBA measured with a sound-level meter at the participants’ position 1 m directly in front of the loudspeaker. An entire test session (for the larger study) took approximately 1 h; the SMRT portion was an adaptive test that typically took around 5 min. The SMRT does not provide practice sessions. However, these were not required as the task instructions were sufficiently clear to be completed by all participants.

#### 2.4.3. Training

The 12 weeks of music training consisted of weekly, 40 min, face-to-face group-based (four to five children) music therapy sessions facilitated by a registered music therapist focused on maximizing access to a broad range of musical skills and activities. More details are in the Lo et al. paper [26], including the curriculum for training. Trainees were also asked to complete preset online music activities using the Music First Junior App [43] 3 times a week for 15–30 min each time. The online music curriculum was developed by the first author, with input from the music therapist to match the group session goals each week. This ‘homework’ was used to complement the group-based session as whilst the latter provided social engagement and interaction with strong ecological validity, the former enabled more analytic, systematic and controlled training simultaneously data logging responses for verification and monitoring.

### 2.5. Statistical Analyses

All analyses were conducted in IBM SPSS Statistics (Version 31). The significance level was set as *p* = 0.05 significance (two-tailed) with Bonferroni corrections for multiple comparisons. Q-Q plots were conducted at baseline which confirmed the data were normally distributed.

For H1: At the first baseline timepoint, a bivariate correlation was conducted to explore which variables were associated with spectral resolution. For the double-baseline analyses (*n* = 12), linear mixed models with restricted maximum likelihood were conducted. The following fixed effects were entered: time (Baseline 1 and Baseline 2), group (1-week retest and 12-week retest/waitlisted cohort), time × group (interaction term), formal music experience, and hearing age (chronological age − age at fitting/implantation). The latter factor failed to converge and was removed from the final model. For the training analyses, participants were entered as random effects with random intercepts. The baseline session results were used as the reference comparison point for the post hoc contrast testing. These models were used to predict changes in spectral resolution over time whilst controlling for previous formal music experience.

For H2: Total scores for formal and informal training were first calculated (as previously described under Section 2.3 Materials). These total scores were then correlated with spectral resolution.

For H2, H3, and exploratory analyses, bivariate correlations were conducted.

## 3. Results

### 3.1. Pre-Training Double-Baseline Testing

Significance testing of the double baselines was conducted to confirm if a learning or practice effect was contributing to any significant changes. Results confirmed there were no statistically significant differences for the main effect of time, *F*(1, 13) = 1.681, *p* = 0.215, or the interaction between time and group, *F*(1, 13) = 0.002, *p* = 0.965. Formal and informal music experience was not a statistically significant factor. These results imply that there was no learning or practice effect for the test materials, nor any effect of natural maturation or development over the 12-week ‘waiting’ time at the start of this study, and hence any subsequent improvement in outcome measures could be better attributed to the music training itself.

### 3.2. Effect of Music Training Intervention on Spectral Resolution (Hypothesis 1)

**Figure 1:**
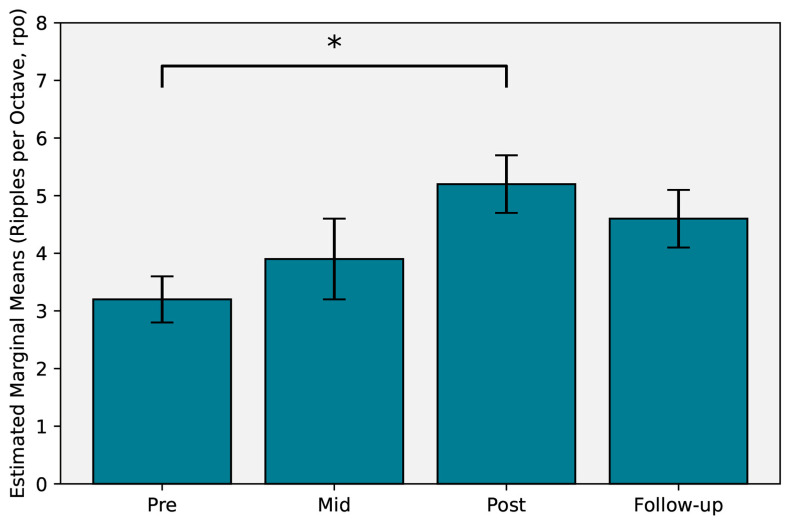
Bar graph of spectral resolution-estimated marginal means across time. Asterisk indicates *p* < 0.05. Error bars represent ± 2 *SE*.

Referenced to the second baseline result, a statistically significant improvement was observed for spectral resolution, measured at the post-training timepoint with a mean increase of 2 rpo, *F*(3, 10.7) = 3.859, *p* = 0.017, *η_p_*^2^ = 0.52, indicating a large partial effect size. However, this improvement was not retained at the follow-up timepoint, with a mean increase of 1.4 rpo, *F*(3, 7.1) = 3.011, *p* = 0.061. Mid-training results were also not significantly different to the second baseline result. Mean scores from the linear mixed model estimates are provided in Table 2 and displayed in Figure 1.

### 3.3. Effect of Prior Formal and Informal Music Engagement on Spectral Resolution (Hypothesis 2)

A summary of the formal and informal music activities undertaken by participants prior to enrolling in the music intervention are reported in Table 3. A correlational analysis was conducted to explore if previous formal and informal music engagement was associated with spectral resolution. Correlations between spectral resolution and both formal (*r* = 0.006, *p* = 0.988) and informal (*r* = 0.007, *p* = 0.986) music engagement demonstrated a non-significant relationship.

### 3.4. Effect of Chronological Age and Hearing Age on Spectral Resolution (Hypothesis 3)

A correlational analysis was conducted to explore if chronological age and hearing age were associated with spectral resolution. Correlations between spectral resolution and both chronological age (*r* = 0.394, *p* = 0.295) and hearing age (*r* = −0.153, *p* = 0.695) demonstrated a non-significant relationship.

### 3.5. Effect of Chronological Age and Hearing Age on Formal and Informal Music Engagement (Exploratory Analyses)

A correlational analysis was conducted to explore if chronological age and hearing age were associated with formal and informal music engagement. Correlations between formal music engagement and both chronological age (*r* = 0.627, *p* = 0.071) and hearing age (*r* = 0.625, *p* = 0.072) demonstrated a positive but non-significant relationship. Correlations between informal music engagement and both chronological age (*r* = 0.390, *p* = 0.299) and hearing age (*r* = 0.208, *p* = 0.592) demonstrated a positive but non-significant relationship.

## 4. Discussion

This study re-analyzed data from a 12-week music-based intervention to examine whether music training was associated with changes in spectral resolution in pediatric CI users. This focus was motivated by limited evidence for developmental change in spectral resolution and the absence of existing intervention-based studies.

### 4.1. Hypothesis 1—Effect of Music Intervention Will Enhance Spectral Resolution Perception

The main finding of this study was that after participation in a 12-week music training program, spectral resolution was significantly enhanced for children with CIs by the post-training timepoint. However, the absence of a retained effect at the follow-up timepoint suggests several possibilities. Firstly, continued engagement with musical training may be necessary for these perceptual benefits to persist or to be consolidated. Secondly, because two participants withdrew from the program which affected assessment at the follow-up timepoint, the lack of a significant statistical result may be reflective of low statistical power rather than true decay. There is support for this given the mean remained elevated relative to baseline. Finally, as the music training materials did not specifically provide the spectral resolution task to learn, it seems reasonable to infer that the benefit is reflective of a true perceptual change rather than a task-learning effect. This is further supported by the finding that there were no changes in task performance for the waitlisted (do-nothing) cohort.

Empirically, our finding aligns with those reported by Aksu et al. [23], who reported that music-integrated phonological awareness training significantly improved spectral resolution (as measured by the SMRT) in a cohort of 23 children with CIs aged 5–8 years. Their training comprised 10 weekly 30 min individual sessions involving exercises targeted at improving a child’s awareness of initial speech sounds, rhymes, and syllables, as well as activities involving sound manipulation, word segmentation, and identification of phonemes. Musical activities such as perceiving melodic contour directions, discriminating between high and low pitches, rhythm tasks and singing tasks were incorporated with the aim of improving a child’s ability to differentiate sound features, participate in rhythm-based activities, and sing along to songs. The authors explicitly stated that they believed the integration of music into the training program was a contributing factor to the improved SMRT scores post training.

A theoretical explanation for these findings is provided by the OPERA hypothesis, which proposes that music training can drive plasticity in auditory processing systems when musical activities place higher perceptual demands on the auditory system than speech [29,30]. Music perception is far more reliant on the capacity to track fine frequency changes compared to speech perception [9]. This conceptualization is also supported by the empirical findings that the number of spectral channels needed to perceive music is greater than that of speech perception in CIs [44]. Within this framework, even with the reduced spectral fidelity inherent to CIs, music training may drive plasticity by encouraging more precise encoding and utilization of the limited spectral cues available, thereby improving sensitivity to spectral differences despite peripheral constraints.

### 4.2. Hypothesis 2—Prior Formal and Informal Music Engagement Will Enhance Spectral Resolution Perception

Contrary to the second hypothesis, levels of formal and informal music participation were not associated with spectral resolution results. This is somewhat surprising given the emerging body of research showing that music involvement, singing, and music education are linked with better pitch perception and singing (i.e., pitch production) skills in children. For example, Welch et al. [45] conducted a pilot study that investigated the impact of weekly classroom-based music lessons held over two terms for 27 children (12 children using HAs and/or CIs, and 17 with NH). The music intervention program had a strong singing component. They found that when compared to pre-intervention scores, the children improved in their singing range, pitch perception and singing competency after the two terms of music classes. Similarly, Torppa et al. [46] recruited 18 children with HL (aged 3–7 years), seven of whom had bilateral CIs, seven with bilateral hearing aids, and four using a bimodal configuration, along with a control group of 20 children with TH. The children with HL participated in a 10-week music intervention program involving weekly 45 min group music sessions. The results showed that children who had participated in more informal music activities outside of the music intervention program had better singing pitch accuracy, although the 10 weeks of music training did not significantly improve singing pitch accuracy levels for these children.

However, several factors may explain why similar findings were not observed in the present study. Firstly, it is highly possible that there were insufficient participants, confounded by high levels of variability in formal and informal music experiences, to demonstrate any significant relationship. Only half of the children had been involved in any formal music training (score > 1.0), and the informal music training scores were wide ranging. It may also be that given the relatively young age of these children, they may not have had enough time to engage with formal and/or informal musical experiences for any resulting benefit to be seen on the SMRT task. Secondly, developmental factors may also have played a role. The perception of the cross-frequency intensity modulation component of spectral resolution matures later than the frequency resolution component [15]. Even for TH children, this skill continues to mature beyond the age of 10 [20], which is older than the children in this study. This, compounded by the fact that many aspects of auditory perception may be delayed or mature at a later chronological age for children with a HL (when compared to a TH child), could mean that the benefits of music training and exposure on spectral resolution may not be seen on the SMRT until an older age. Appropriately powered longitudinal studies examining how musical engagement interacts with auditory development across childhood may help clarify these relationships.

### 4.3. Hypothesis 3—Effect of Chronological Age and Hearing Age on Spectral Resolution

This latter point may also explain the non-significant finding for Hypothesis 3, as neither chronological age nor hearing age were associated with spectral resolution perception. The children in this study had a mean chronological age of 7.3 years (range: 6–9.2 years) and a mean hearing age of 6.3 years (range: 3.1–8.4). Hence, if spectral resolution for a TH child matures after the age of 10 and into adolescence [3,19,20], it would be reasonable to assume that this skill was yet to mature in the children in our study, coupled with a sample size that was insufficient in showing any statistically significant developmental trajectory. This would have been further impacted by the spectral smearing associated with electrical stimulation from the CI [12], along with the psychoacoustic deficits associated with significant sensorineural hearing losses [47]. Future research should investigate what the critical developmental milestones and ages are regarding the developmental trajectory for spectral and frequency resolution in children with HL.

### 4.4. Exploratory Analyses—Effect of Chronological Age and Hearing Age on Formal and Informal Music Engagement

Our findings did not support chronological or hearing age being associated with formal and informal music engagement. Similarly, the young age of this cohort (i.e., limited time to participate in music activities), limited age range, along with the small sample size would likely have been the primary driver of this non-significant finding. The hearing age of the children in this cohort was also similarly limited with a mean of 6.3 years (range: 3.1–8.4). It is also worthwhile pointing out that it would not be unreasonable to postulate that for most children with HL, the initial months/years after being fitted with their hearing device is spent focused on speech and language development, with music being less of a priority, and therefore music exposure in these early developmental years may be limited.

In the original study that used the RMFQ to examine the role of music in families of children with HL (*n* = 56) and TH (*n* = 322) aged between 2 and 6 years, the authors also found no significant correlation between age (chronological age, age diagnosed with HL and/or age first fitted with their hearing device) and overall music participation (formal and informal grouped together), overall music enjoyment, the importance of music in the family’s life, the importance of music in the child’s life, and/or their child’s love of music [41].

The authors proposed that family musical environment and opportunities for musical exposure may play a more influential role than age in shaping young children’s musical experiences [41].

Finally, whilst not statistically significant, both age and music engagement showed a positive association (i.e., older children tended to have greater levels of music engagement). Again, this null finding likely resulted from being statistically underpowered; having a limited age range of participants; as well as the young children having limited access/opportunity to engage with music, due to their young age.

### 4.5. Limitations and Future Directions

This study has several limitations to note and consider. Firstly, this study had a small cohort extracted from an existing data set to examine the specificity of CI and bimodal configurations, relative to the original study that had a more heterogenous cohort with HL. Indeed, a larger cohort of children across a wider range of ages will be required to examine critical development milestones in respect to spectral resolution (and other auditory outcomes of interest), and potentially reduce some of the inherent biases that may be present with small, self-selecting participant groups. The use of a randomized controlled trial design with an active intervention for the control group will also be able to provide more definitive conclusions. It should also be noted that the impact of the small sample size was greater for analyses relevant to H2 and H3. The use of repeated measures for the statistical analysis of H1 provided more statistical power when compared to the correlational analyses for H2 and H3.

An additional consideration is the inherent heterogeneity with CIs. Factors such as variability in neural integrity, cognition, and device-related characteristics (e.g., processor type and signal-processing strategies) were not explicitly controlled in the present study and may contribute to individual differences in spectral resolution. However, this variability also reflects the diversity of real-world CI users and, as such, enhances the ecological validity of the findings. The present study was designed to capture broad behavioral outcomes and future research incorporating more detailed characterization of neural and device-related factors would be valuable in future studies. An open question that remains is the potential role of cognition in relation to music training and spectral resolution.

The key finding was that spectral resolution could be enhanced through music training, with the OPERA hypothesis providing a plausible mechanism to explain this finding. Furthering our understanding of both spectral resolution development and whether auditory interventions could improve spectral acuity may lead to the development of more effective (re)habilitation strategies to support the auditory development of children with HL, as well as a more comprehensive understanding of mechanisms that underlie intervention-based perceptual gains. For example, is it the child’s ability to focus on important spectral information that has improved? Alternatively, is it that the cues become more salient to them and therefore the improvement is a result of the child being better able to detect and differentiate these frequency-based or fine-structure cues, consolidate them and subsequently effectively use the cues? On the other hand, are they simply more sensitive to intensity modulations and better able to compare intensity cues across frequencies and perceive difference in the intensity of the spectral component of the signal?

A 12-week intervention period consisting of weekly music training has been shown to be sufficient to result in measurable perceptual benefits (such as improvements to spectral resolution perception), but longer-term interventions that facilitate sustained benefits would likely generate greater transferable, wide-reaching benefits for children with CIs. A recent meta-analysis on music training studies for children aged 3–11 years incorporating a control group reported that music training helped to improve executive functioning, specifically inhibition control, which subsequently impacted on language tasks [48]. Putkinen at al. [49] found that informal musical activity was associated with a more mature auditory attention and better ability to suppress distracting sounds, measured with attention-related brain responses (P3as). These results, in conjunction with the preliminary findings from our study, indicate that it would also be valuable to assess the associations between SMRT, music training and higher-order cognitive processes in a larger randomized control trial.

## 5. Conclusions

The main finding of this study was that 12 weeks of music training was sufficient to result in improved spectral resolution perception for children with CIs. This suggests that despite the known limitations of CI technology on spectral resolution, music training is a potentially effective intervention to improve spectral resolution perception in children using CIs, in addition to all the other benefits music has been shown to provide in the existing research. Given that spectral resolution is closely linked to speech perception outcomes, these findings further highlight the clinical relevance of music-based rehabilitation.

## Figures and Tables

**Table 1:** Demographic information of participants.

ID	Group ^1^	Age/ Hearing Age (Yrs) ^2^	Age at First Fitting of HA or CI (Yrs) ^3^	Sex	Formal Music Experience ^4^	Informal Music Experience ^4^	Degree of HL	Device Configuration
HL1	1	6.3/6.0	0.3 ^†^	F	0	7.6	L: Profound; R: Profound	CI
HL3	1	8.3/7	1.3	M	3.7	7.7	L: Profound; R: Profound	CI
HL5	1	6.1/3.1	3.0	F	0.7	4.6	L: Profound; R: Moderate	Bimodal
HL6	1	7.8/7.5	0.3	M	1.3	8.7	L: Moderately severe; R: Severe	Bimodal
HL11	2	6.7/6.2	0.5	F	0	2.1	L: Moderately severe; R: Profound	Bimodal
HL12	2	7.8/5.8	2.0	M	4.3	17	L: Profound; R: Profound	CI
HL14	2	6.7/4.9	1.8	F	0.2	2.2	L: Profound; R: Profound	CI
HL15	2	6.3/6.0	0.3 ^†^	M	1.3	11.9	L: Profound; R: Profound	CI
HL17	2	6.8/6.7	0.1 ^†^	F	4.5	4	L: Profound; R: Severe	Bimodal
HL18 *	2	9.2/7.2	2.0	F	0	0	L: Profound; R: Profound	CI
HL19 *	2	6.8/6.3	0.5	M	0	6.1	L: Profound; R: Profound	CI
HL20 *	2	8.8/8.4	0.4	M	3.6	0.2	L: Profound; R: Profound	CI
Mean	NA	7.3/6.3	1.0	NA	1.6	6.0	NA	NA

* Only completed baseline testing. ^1^ Group 1 participants were enrolled into the music program straight away while Group 2 participants were waitlisted before the music program. ^2^ Measured at the commencement of music training, calculated as ‘chronological age’ minus ‘age at fitting/implantation’. ^3^ First fitting of a HA or CI, noting that some participants transitioned from a HA to a CI and are denoted with a ^†^. ^4^ Calculated from the RMFQ data, as explained in Section 2.3.

**Table 2:** Spectral resolution-estimated marginal means across time.

Time	Estimate (*M*, *SE*)	*t*	*p*	95% Confidence Interval	
				Lower Bound	Upper Bound
Pre	3.2 (0.4)	-	-	2.3	4.1
Mid	3.9 (0.7)	1.172	0.815	2.5	5.4
Post	5.2 (0.5)	3.859	0.017	3.9	6.4
Follow-up	4.6 (0.5)	3.011	0.061	3.4	5.8

**Table 3:** Formal and informal music activities.

	Activity Type	Participating Participants, *n* (%) *	Frequency (*M*, *SD*)	Duration (*M*, *SD*)	Formal or Informal Score (*M*, *SD*)
**Formal Music Activities**	Music lessons	5 (42%)	1.67 (2.06)	0.71 (1.05)	0.47 (0.70)
	Singing groups	5 (42%)	1.58 (2.35)	0.75 (1.06)	0.53 (0.86)
	Dance classes	4 (33%)	1.50 (2.28)	0.58 (0.90)	0.42 (0.62)
	Instrumental groups	2 (17%)	0.67 (1.65)	0.13 (0.43)	0.08 (0.21)
	Special children’s programs	1 (8%)	0.33 (1.15)	0.13 (0.43)	0.08 (0.29)
	Other music programs	1 (8%)	0.33 (1.15)	0.17 (0.58)	0.11 (0.38)
**Informal Music Activities**	Listening to music informally	9 (75.0%)	4.50 (2.88)	3.29 (2.97)	2.19 (2.10)
	Musical videos	8 (66.7%)	3.33 (2.65)	1.42 (1.44)	0.69 (0.80)
	Dancing informally	8 (66.7%)	3.33 (2.84)	1.29 (1.18)	0.67 (0.64)
	Social music activities	7 (58.3%)	1.50 (2.27)	1.79 (2.29)	0.47 (0.75)
	Creating/making music during play	7 (58.3%)	2.92 (2.63)	1.71 (1.43)	0.73 (0.88)
	Independent musical exploration	6 (50.0%)	1.67 (2.14)	1.79 (2.43)	0.73 (1.43)
	Music concerts	6 (50.0%)	0.50 (0.52)	2.17 (2.60)	0.24 (0.29)
	Family music activities	3 (25.0%)	0.75 (1.90)	0.33 (0.65)	0.08 (0.20)
	Online music training or games	1 (8.3%)	0.42 (1.44)	0.33 (1.15)	0.19 (0.64)

* Note that this does not add up to 100% as children could participate in more than one activity.

## Data Availability

The raw data supporting the conclusions of this article will be made available by the authors on request.

## Abbreviations

The following abbreviations are used in this manuscript:

CI	Cochlear implant
HL	Hearing loss
SIN	Speech-in-noise
TH	Typical hearing
SMRT	Spectral-Temporally Modulated Ripple Test
rpo	Ripples per octave
RMFQ	The Role of Music in Families Questionnaire
